# Soluble Fibrinogen–Like Protein 2 Downregulation and Th17/Treg Imbalance in a Taurocholate‐Induced Murine Experimental Model of Severe Acute Pancreatitis

**DOI:** 10.1002/jcla.25076

**Published:** 2024-06-09

**Authors:** Yibing Hu, Jin Ding, Yanping Chen, Qunying Wang, Xiaoyun Yang, Hongjun Hua, Xiaohua Ye

**Affiliations:** ^1^ Department of Gastroenterology, Affiliated Jinhua Hospital Zhejiang University School of Medicine Jinhua Zhejiang China

**Keywords:** immunomodulation, inflammation, regulatory T cell, severe acute pancreatitis, soluble fibrinogen–like protein 2, Th17/Treg imbalance, T‐helper 17 cell

## Abstract

**Background:**

Severe acute pancreatitis (SAP) is associated with tremendous systemic inflammation, T‐helper 17 (Th17) cells, and regulatory T (Treg) cells play an essential role in the inflammatory responses. Meanwhile, soluble fibrinogen–like protein 2 (Sfgl2) is a critical immunosuppressive effector cytokine of Treg cells and modulates immune responses. However, the impact of SAP induction on Sfgl2 expression and the role of Sfgl2 in immunomodulation under SAP conditions are largely unknown.

**Methods:**

A taurocholate‐induced mouse SAP model was established. The ratios of CD4^+^CD25^+^Foxp3^+^ Treg cells or CD4^+^IL‐17^+^ Th17 cells in blood and pancreatic tissues as well as surface expression of CD80, CD86, and major histocompatibility complex class II (MHC‐II) were determined by flow cytometry. Gene mRNA expression was determined by qPCR. Serum amylase and soluble factors were quantitated by commercial kits. Bone marrow–derived dendritic cells (DCs) were generated, and NF‐κB/p65 translocation was measured by immunofluorescence staining.

**Results:**

SAP induction in mice decreased the Th17/Treg ratio in the pancreatic tissue and increased the Th17/Treg ratio in the peripheral blood. In addition, SAP was associated with a reduced level of Sfgl2 in the pancreatic tissue and blood: higher levels of serum IL‐17, IL‐2, IFN‐α, and TNF‐α, and lower levels of serum IL‐4 and IL‐10. Furthermore, the SAP‐induced reduction in Sfgl2 expression was accompanied by dysregulated maturation of bone marrow–derived DCs.

**Conclusions:**

SAP causes reduced Sfgl2 expression and Th17/Treg imbalance, thus providing critical insights for the development of Sfgl2‐ and Th17/Treg balance‐targeted immunotherapies for patients with SAP.

## Graphical abstract

SAP induction in mice lowered the Th17/Treg ratio in pancreatic tissues and increased the ratio of Th17/Treg in the peripheral blood. SAP was associated with a reduced level of Sfgl2 in the pancreatic tissue and blood, as well as altered levels of soluble factors including IL‐17, IL‐2, IFN‐α, TNF‐α, IL‐4, and IL‐10. The SAP‐induced reduction in Sfgl2 expression was accompanied by dysregulated maturation of bone marrow–derived DCs.

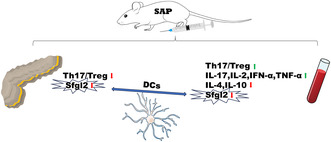

## 1 Introduction

As a common gastrointestinal disease, acute pancreatitis (AP) has an increasing incidence and is one of the most frequent gastrointestinal causes of hospital admissions [1, 2]. Approximately 15% of patients with AP develop severe acute pancreatitis (SAP), which is associated with high morbidity and mortality rates [3, 4, 5], largely due to inflammation of the exocrine pancreas, acinar cell injury, extrapancreatic necrosis, and potential multisystem organ failure (MOF) [6, 7]. The underlying mechanisms of SAP involve a complex cascade of events, such as inflammation, microcirculatory disturbances, and coagulation abnormalities, thus making the etiology of SAP heterogeneous [8, 9]. SAP includes persistent systemic inflammatory response syndrome (SIRS) and MOF; however, the mechanisms underlying the transition from SIRS to MOF are largely unknown, although some inflammatory factors have been proposed to be involved in the process [10]. For example, tumor necrosis factor alpha (TNF‐α), one of the most important cytokines in AP pathogenesis, is considered as a reliable factor for predicting the severity and outcome of SAP. It has been reported that a low TNF‐α level in patients with SAP can predict the development of multiple organ dysfunction syndrome and a worse prognosis [11]. Because of the limited understanding of the pathogenesis of SAP and the rather complicated causality of this disease, the underlying pathophysiological mechanisms of SAP remain incompletely elucidated and few effective treatment modalities have been developed [12, 13]. Therefore, the identification of SAP‐related biomarkers and therapeutic targets is urgently needed for the effective management of SAP and its complications.

Accumulating research indicates that various inflammatory cells and cytokines contribute to the development of AP, and a series of complex immune events are involved in its progression [14]. Immunomodulatory therapy regulates the imbalance of inflammatory responses, attenuates SAP‐related tissue injuries, and improves the outcomes of patients with SAP [15]. Thus, monitoring the parameters and levels of inflammatory mediators associated with immune cells to develop immunomodulatory therapy might help to improve the outcomes of patients with SAP. As a unique cluster of differentiation (CD)4^+^ T‐helper subset, T‐helper 17 (Th17) cells are featured with the production of interleukin (IL)‐17, which accelerates tissue inflammation [16, 17]. As a specialized lineage of CD4^+^ suppressor T cells, regulatory T (Treg) cells function as an essential suppressor of inflammation under various physiological and pathological conditions [18]. The imbalance of Th17/Treg has been identified in patients with SAP and has been proposed to be correlated with SAP severity and the prognosis of patients [19]. Thus, it is paramount to elucidate the molecular mechanisms underlying the pathogenesis of the Th17/Treg imbalance in the development and treatment of patients with SAP.

Fibrinogen‐like protein 2 (Fgl2) is a member of the fibrinogen‐associated protein superfamily; it plays essential roles in various normal biological contexts as well as the pathogenesis of various diseases, such as infectious inflammation, autoimmune disorders, and tumorigenesis [20]. Fgl2 is presented as two structurally distinct forms: membrane‐bound Fgl2 (Fgl2) and soluble Fgl2 (Sfgl2). Fgl2 is predominantly expressed on reticuloendothelial cells and exerts procoagulant activity, whereas Sfgl2 is constitutively expressed by T cells and demonstrates contradictory functions in the process of tissue damage [21]. Sfgl2 acts as a critical immunosuppressive effector and soluble factor of Treg cells, and it can suppress the production of T helper 1 (Th1) cytokines while enhancing Th2 cytokines [20, 22]. Moreover, Sfgl2 has been reported to inhibit T‐cell proliferation, suppress dendritic cell (DC) maturation, enhance M2 macrophage polarization, and promote B‐cell apoptosis [20, 23]. Previously, we have revealed that Fgl2 expression is increased in both the SAP rat model and patients with SAP, and a positive correlation between human Fgl2 expression in the blood cells of patients and the severity of SAP has been identified [24, 25, 26]. However, the impact of SAP induction on the expression of Sfgl2 and the role of Sfgl2 in immunomodulation are largely unknown.

In the current study, we established a taurocholate‐induced murine experimental model of SAP and evaluated how SAP induction affects the balance of Th17/Treg through analyzing the percentages of Treg cells and Th17 cells in the peripheral blood and pancreatic tissue. In addition, the changes in the concentrations of serum Sfgl2 and other serum‐soluble inflammation‐related factors in SAP mice as well as the potential role of Sfgl2 in modulating DC maturation during SAP induction were explored.

## 2 Methods

### 2.1 Animals

Female C57BL/6J mice (6–8 weeks old, *n* = 100) were purchased from Research Science Biotechnology Co. Ltd. (Shanghai, China) and kept in specific‐pathogen‐free conditions at the Laboratory Animal Center of Risai Biomedical Technology Co., Ltd. (Zhejiang, China). The mice were housed in an animal room with a 12‐h alternating light–dark cycle and had free access to sufficient food and water. All animals were acclimated for at least 7 days before the start of the experiments. All experiments involving the use of animals were approved by the Experimental Animal Ethics Committee of Risai Biomedical Technology Co., Ltd. (approval No. 20201110012) and were carried out in accordance with the Guide for the Care and Use of Laboratory Animals (8th edition) from the National Research Council (US) Committee. All methods were reported in accordance with the ARRIVE guidelines (https://arriveguidelines.org) for the reporting of animal experiments.

### 2.2 SAP Experimental Modeling

The mouse model of sodium taurocholate‐induced SAP was established according to methods described previously [24]. Briefly, the mice were first anesthetized through intraperitoneal injection of 10% chloraldurate (2 mL/kg body weight; Solarbio, Beijing, China). The mice were then divided into two groups: sham operation (Sham) group and SAP induction (SAP) group. The mice in the SAP group received a laparotomy through a midline incision and were retrogradely injected with sodium taurocholate (5%; 1 mL/kg body weight; Sigma, St. Louis, MO, USA) into the biliopancreatic duct. The mice in the Sham group underwent surgery but were not infused with 5% sodium taurocholate. The mouse abdomen was closed in two layers, and all surgical processes were conducted with stringent sterile techniques.

### 2.3 Blood and Pancreas Sample Collection

At 4 h after SAP induction or the sham operation, the mice were anesthetized with 10% chloraldurate (2 mL/kg body weight) and euthanized by exsanguination. Blood samples (approximately 1.5 mL) were obtained via a postcava puncture. Half of the blood sample in a 1.5‐mL tube without anticoagulants was centrifuged at 12,000 × *g* for 10 min, and the serum was collected for determining the concentrations of Sfgl2 and other cytokines as well as amylase activity. The remaining blood sample was stored in ethylene diamine tetraacetic acid (EDTA)‐containing tubes (Beikete Company, Wenzhou, China) and subjected to the isolation of peripheral blood mononuclear cells (PBMCs) with density gradient centrifugation using Ficoll solution (Solarbio, Beijing, China), according to the manufacturer's instructions. The pancreatic tissues were harvested immediately after the mice were sacrificed. Small portions of the tissues were fixed in 4% paraformaldehyde for histological analysis, and some large portions were used for flow cytometric analysis after dissociation with trypsin–EDTA digestion. Other portions of pancreatic tissues were stored in liquid nitrogen until use for gene expression quantitation.

### 2.4 Flow Cytometry

The ratios of CD4^+^CD25^+^Foxp3^+^ Treg cells or CD4^+^IL‐17^+^ Th17 cells among the total population of CD4^+^ T cells in PBMCs and pancreatic tissues were determined by flow cytometry, according to the manufacturer's protocol. For staining of Treg cells, the collected cells were stained with fluorescein isothiocyanate (FITC)‐conjugated anti‐mouse CD4 antibody and allophycocyanin (APC)‐conjugated anti‐CD25 antibody (BioLegend, San Diego, CA, USA). After surface staining, the T cells were fixed, permeabilized, and further stained with phycoerythrin (PE)‐conjugated anti‐mouse Foxp3 antibody (BioLegend). For staining of Th17 cells, the cells were incubated with FITC‐conjugated anti‐mouse CD4 antibody (BioLegend). After fixation and permeabilization, the cells were stained with PE‐conjugated anti‐mouse IL‐17 antibody (BioLegend). For staining of DCs, the cells were stained with PE‐conjugated anti‐mouse CD80, CD86, and major histocompatibility complex class II (MHC‐II) antibodies (BioLegend), separately. All staining processes were performed at 4°C for ~30 min, and the cells were washed with phosphate‐buffered saline (PBS) supplemented with 2% fetal bovine serum (FBS; Sigma) after each staining. Data were acquired with a BD FACSCalibur cytometer and were analyzed using FlowJo software (BD Biosciences).

### 2.5 Histological Analysis

The pancreatic tissues were dissected, fixed in 4% paraformaldehyde, and decalcified in 10% formic acid at room temperature. Some 4‐μm serial sections were stained with hematoxylin and eosin (H&E), according to the standard protocol, to evaluate the histopathological changes caused by SAP induction. Histological observations of H&E‐stained slides and single cells (dissociated from pancreatic tissues and DCs differentiated from bone marrow) were conducted from photographs captured under an optical microscope (E100 microscope; Nikon Instruments, Tokyo, Japan).

### 2.6 Real‐Time Quantitative Polymerase Chain Reaction (qPCR)

Total RNA was extracted from pancreatic tissues using TRIzol reagent (Thermo Fisher Scientific, Waltham, MA, USA), according to the manufacturer's instructions. The synthesis of cDNA was conducted by a RevertAid RT Reverse Transcription Kit (Thermo Fisher Scientific). Reverse transcription qPCR (RT‐qPCR) was conducted using the standard protocol for the use of SYBR Green qPCR Master Mix (Thermo Fisher Scientific) on an AB7500 RT‐PCR instrument (Applied Biosystems, Foster City, CA, USA). The sequences of PCR primers used in this study are as follows: *Foxp3*, forward, 5′‐CTCCTACCCCACTGCTGGCAAAT‐3′, reverse, 5′‐CCCTGCCCTTCTCATCCAGA‐3′; *Sfgl2*, forward, 5′‐TCTGGGAACTGTGGGCTCTA ‐3′, reverse, 5′‐GGAGGACTTGTAGCCACCTG‐3′; *Gapdh*, forward, 5′‐AAGAAGGTGGTGAAGCAGG‐3′, reverse, 5′‐GAAGGTGGAAGAGTGGGAGT‐3′. The qPCR experiments were conducted with each sample run in triplicate. The 2^−ΔΔCT^ method with normalization to the housekeeping gene *Gapdh* was used to calculate the expression levels of target genes.

### 2.7 Determination of Serum Amylase

Blood samples were collected, and the subsequently separated serum was preserved at −80°C for further assays. The serum amylase activity was determined using an AMS test kit (Jiancheng Bioengineering, Nanjing, China), according to the manufacturer's instructions. The enzyme activity was expressed in units per deciliter of serum (U/dL), as reported previously [27].

### 2.8 Enzyme‐Linked Immunosorbent Assay (ELISA)

The serum level of Sfgl2 was detected in duplicate using an ELISA kit (BioLegend, San Diego, CA, USA), as described previously [28, 29]. The serum levels of IL‐2, IL‐4, IL‐10, IL‐17, interferon alpha (IFN‐α), and TNF‐α were measured by ELISA using kits from Beyotime Biotechnology (Shanghai, China). All assays were performed according to the manufacturer's instructions.

### 2.9 Generation of Bone Marrow–Derived DCs

Bone marrow–derived DCs were generated as previously reported [30]. Briefly, on Day 0, the bone marrow was flushed out of the femurs and tibias of the sham and SAP mice into Hanks' Balanced Salt Solution supplemented with 2% FBS using a 24G needle. The bone marrow was pipetted vigorously to make a single cell suspension and then passed through a cell strainer (70‐μm nylon mesh; BD Falcon, Franklin Lakes, NJ, USA). The bone marrow cells were washed twice with complete culture medium (RPMI 1640 supplemented with 10% FBS, 1% penicillin and streptomycin, and 1× GlutaMax; all from Thermo Fisher Scientific) and cultured at 1 × 10^6^ cells/well in a 6‐well plate in 6 mL of complete medium supplemented with 3 ng/mL IL‐4 and 6 ng/mL granulocyte‐macrophage colony‐stimulating factor (GM‐CSF) for 6 days at 37°C in an incubator with 5% CO_2_. Half of the medium was replaced on Days 3–5 with fresh prewarmed complete culture medium supplemented with 3 ng/mL IL‐4 and 6 ng/mL GM‐CSF. On Day 6, the loosely adherent cells were harvested as bone marrow–derived DCs and further purified using a MagniSort Mouse CD11c Positive Selection Kit (Miltenyi Biotech, North Rhine‐Westphalia, Germany) for DC activation assays.

### 2.10 Immunofluorescence Staining

Harvested DCs generated from the Sham and SAP mice were allowed to adhere to autoclaved glass coverslips overnight at 37°C/5% CO_2_ and incubated in complete medium supplemented with 10% FBS, 3 ng/mL IL‐4, and 6 ng/mL GM‐CSF. Nuclear factor kappa B (NF‐κB)/p65 translocation was examined the next day after seeding the DCs on the glass coverslips. After cell fixation using PBS supplemented with 2% paraformaldehyde for 30 min, the coverslips were washed three times with PBS, permeabilized with 0.2% Triton X‐100 in PBS, and then blocked with 5% bovine serum albumin in PBS for 30 min at room temperature. Then, the slides were incubated with a goat anti‐p65 polyclonal antibody (1:100 dilution; Abcam, USA) for 40 min at room temperature. After washing with PBS, the slides were further incubated with FITC‐conjugated donkey anti‐goat IgG secondary antibody (1:500 dilution; Biosharp, Anhui, China) for 1 h at room temperature. The nuclei were counterstained with 4′,6′‐diamidino‐2‐phenylindole chromosomal staining (Molecular Probes, Eugene, OR, USA). The slides were observed, and images were taken using a fluorescence microscope (Olympus, Japan).

### 2.11 Statistical Analysis

Data are expressed as the mean ± standard deviation, and groups were compared using the unpaired, two‐tailed Student's *t*‐test. The Mann–Whitney test was used to determine statistically significant changes in variables that were not normally distributed. Statistical analyses were performed using SPSS, version 19.0, statistical software (IBM Corporation; Armonk, NY, USA), and data were plotted with GraphPad Prism (version 8.0; GraphPad Software, Inc., San Diego, CA, USA). In all cases, a *p‐*value < 0.05 was considered statistically significant.

## 3 Results

### 3.1 Histological and Cellular Analyses Confirmed the Successful Establishment of Taurocholate‐Induced SAP in Mice

**FIGURE 1:**
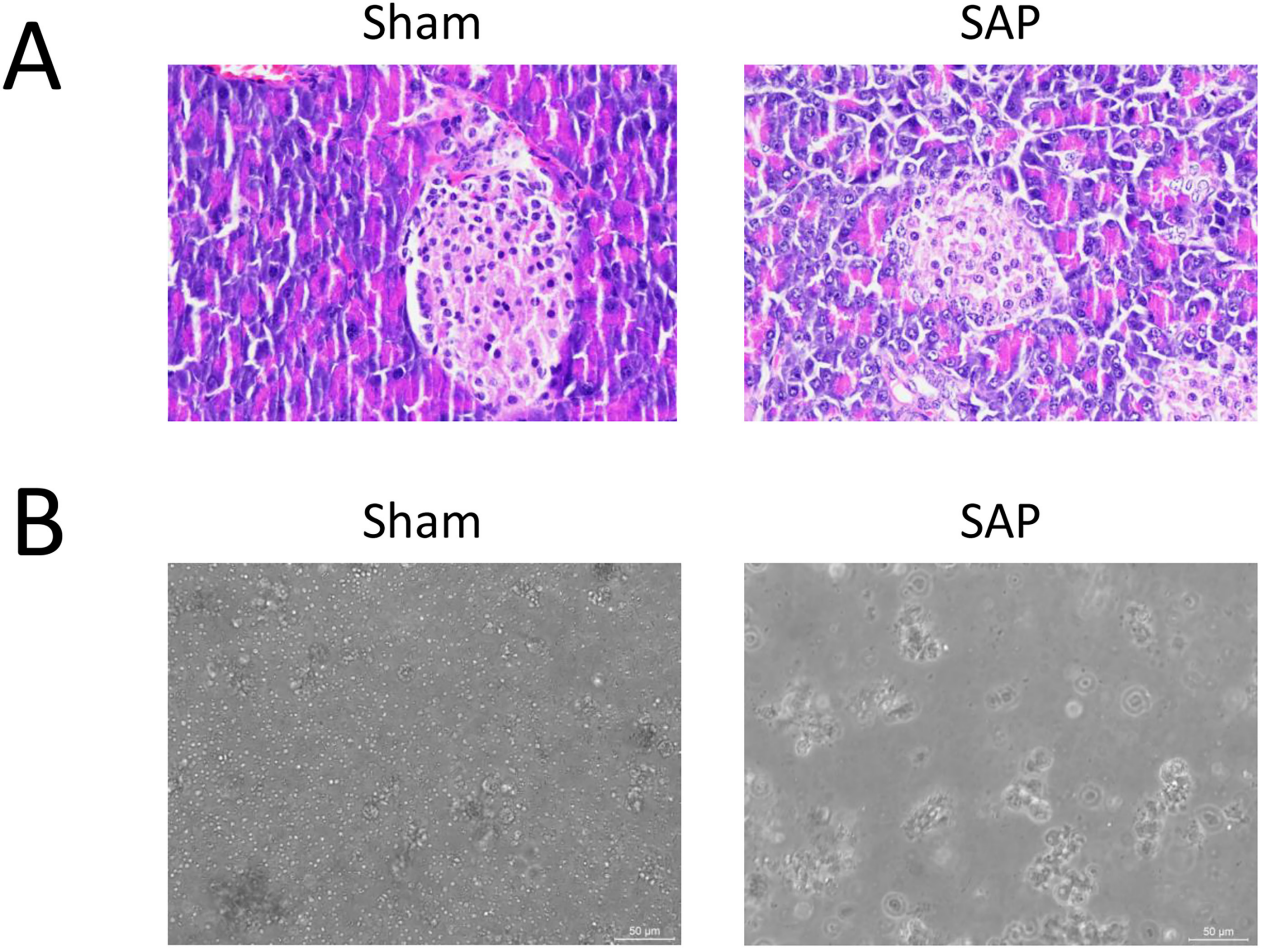
Histological and cellular analyses confirmed that sodium taurocholate administration induced SAP in mice. (A,B) After anesthesia, the mice were divided into two groups: The sham operation (Sham) group and the SAP induction (SAP) group. The SAP mice were retrogradely injected with sodium taurocholate (5%; 1 mL/kg body weight) into the biliopancreatic duct, and the sham mice underwent surgery without sodium taurocholate infusion. At 4 h after the SAP induction or sham operation, the mice were anesthetized and euthanized. The pancreatic tissues were harvested immediately for histological analysis and dissociated for cellular analysis. (A) Representative images showing the histological changes in pancreatic tissues by H&E staining. Magnification, 400×. (B) Representative images showing the cellular morphological changes in dissociated pancreatic cells. Magnification, 20×.

To validate that the administration of taurocholate did indeed induce SAP in our current experimental setting, we first examined the histopathological changes in the pancreatic tissues from the Sham and SAP mice. The histological features of the pancreas in the Sham group seemed morphologically normal, whereas the pancreatic tissues in the SAP group appeared evidently damaged. In addition, microscopic examination of the H&E‐stained slices showed that the pancreas in the SAP group had much more edema, hemorrhage, acinar cell necrosis on the pancreas mucosa, and inflammation with neutrophil infiltration than that in the Sham group (Figure 1A). Moreover, the dissociated single cells from the pancreas of the SAP group exhibited obviously more large‐sized vacuole‐like cell clusters than those from the Sham group (Figure 1B), indicating that taurocholate induced a potentially low viability of pancreatic cells and widespread pancreatic tissue injury. Therefore, the evidence from these imaging data clearly suggests that the SAP murine model with sodium taurocholate‐induced histopathological alterations of the pancreas was established successfully.

### 3.2 The SAP Murine Model Displayed Th17/Treg Imbalance in Blood and Pancreas Samples

**FIGURE 2:**
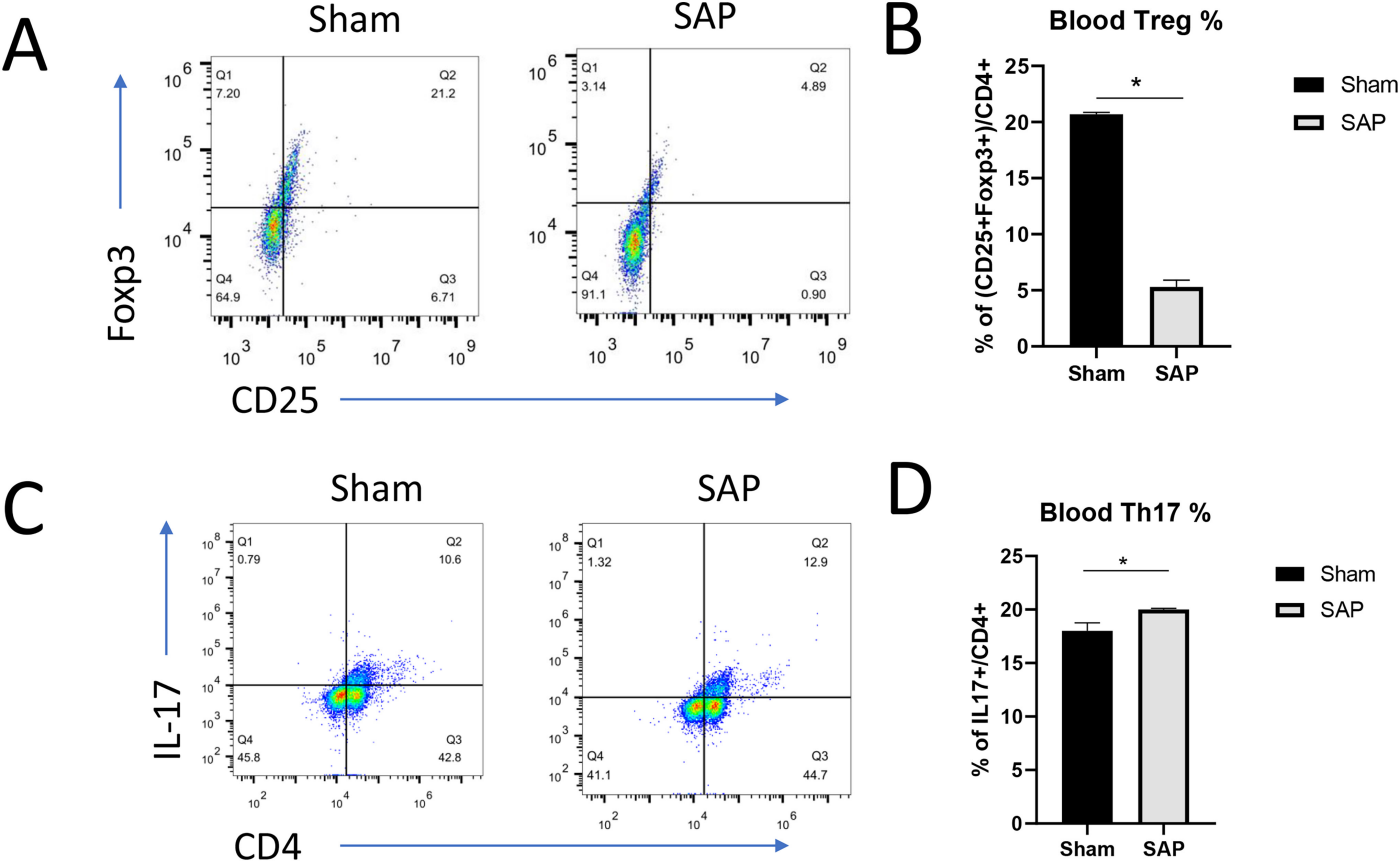
SAP induction changed the frequencies of Treg cells and Th17 cells in mouse peripheral blood. (A–D) At 4 h after surgery, the frequencies of CD25^+^Foxp3^+^ Treg cells and CD4^+^IL‐17^+^ Th17 cells among the total CD4^+^ T cells in blood samples from the Sham mice and SAP mice were measured by flow cytometry. Representative profiles showing the percentages of CD25^+^Foxp3^+^ Treg cells (A) and CD4^+^ IL‐17^+^ Th17 cells (C) in blood samples from the Sham and SAP groups. The percentages of blood Treg cells (B) and Th17 cells (D) are summarized. *n* = 5 mice per group, *p < 0.05, between the indicated groups.

**FIGURE 3:**
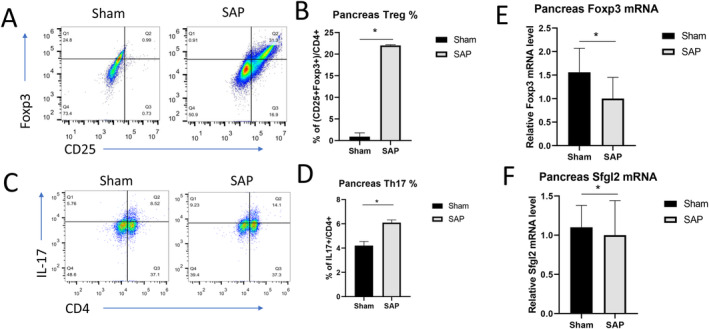
SAP induction changed the frequencies of Treg cells and Th17 cells in mouse pancreatic tissues. (A–D) At 4 h after surgery, the frequencies of CD25^+^Foxp3^+^ Treg cells and CD4^+^IL‐17^+^ Th17 cells among the total CD4^+^ T cells in pancreatic tissues from the sham mice and SAP mice were measured by flow cytometry. Representative profiles showing the percentages of CD25^+^Foxp3^+^ Treg cells (A) and CD4^+^ IL‐17^+^ Th17 cells (C) in pancreatic tissues samples from the Sham and SAP groups. The percentages of Treg cells (B) and Th17 cells (D) in the pancreatic tissues are summarized. (E, F) The mRNA levels of *Foxp3* (E) and *Sfgl2* (F) in the pancreatic tissues were quantitated by qPCR. *n* = 5 mice per group, *p < 0.05, between the indicated groups.

To investigate the potential involvement of the Th17/Treg imbalance in the pathogenesis of SAP, we examined the percentages of CD4^+^CD25^+^Foxp3^+^ Treg cells and CD4^+^IL‐17^+^ Th17 cells in PBMCs and pancreatic tissues isolated from the Sham mice and the SAP mice. As shown in Figure 2A,B, the peripheral blood from the mice in the Sham group displayed ~20% CD25^+^Foxp3^+^ Treg cells among all CD4^+^ T cells, whereas blood from the SAP group had only ~5% CD25^+^Foxp3^+^ Treg cells. On the contrary, the CD4^+^ T cells in the blood from the SAP group seemed to contain a significantly higher percentage of CD4^+^IL‐17^+^ Th17 cells than those from the Sham group (Figure 2C,D). Thus, SAP induction in our murine model remarkably increased the Th17/Treg ratio in the peripheral blood cells. However, we found that the pancreatic tissues from the SAP group showed a markedly higher percentage of CD4^+^CD25^+^Foxp3^+^ Treg cells among the total CD4^+^ T cells than the Sham group (Sham vs. SAP, ~1% vs. ~30%; Figure 3A,D). In addition, the pancreatic tissues from the SAP mice only showed very slightly increased percentages of CD4^+^IL‐17^+^ Th17 cells compared with those from the Sham mice (Sham vs. SAP, ~9% vs. ~14%; Figure 3C,D). Therefore, SAP induction profoundly reduced the Th17/Treg ratio in pancreatic tissues in our murine model. Further qPCR analyses demonstrated that the pancreatic tissues in the SAP group had a slightly decreased mRNA level of *Foxp3* (Figure 3E), implicating a potentially transient accumulation of Treg cells in pancreatic tissues upon SAP induction whereas the absolute number of total Treg cells was still low in the pancreas. Moreover, the SAP group also had a slightly lower level of *Sfgl2* mRNA in the pancreatic tissues than the Sham group (Figure 3F), suggesting a potential link between Sfgl2 and regulation of the Th17/Treg imbalance in SAP pathogenesis.

### 3.3 SAP Induction Led to a Reduced Sfgl2 Concentration and Altered Levels of Inflammatory Mediators in Mouse Serum

**FIGURE 4:**
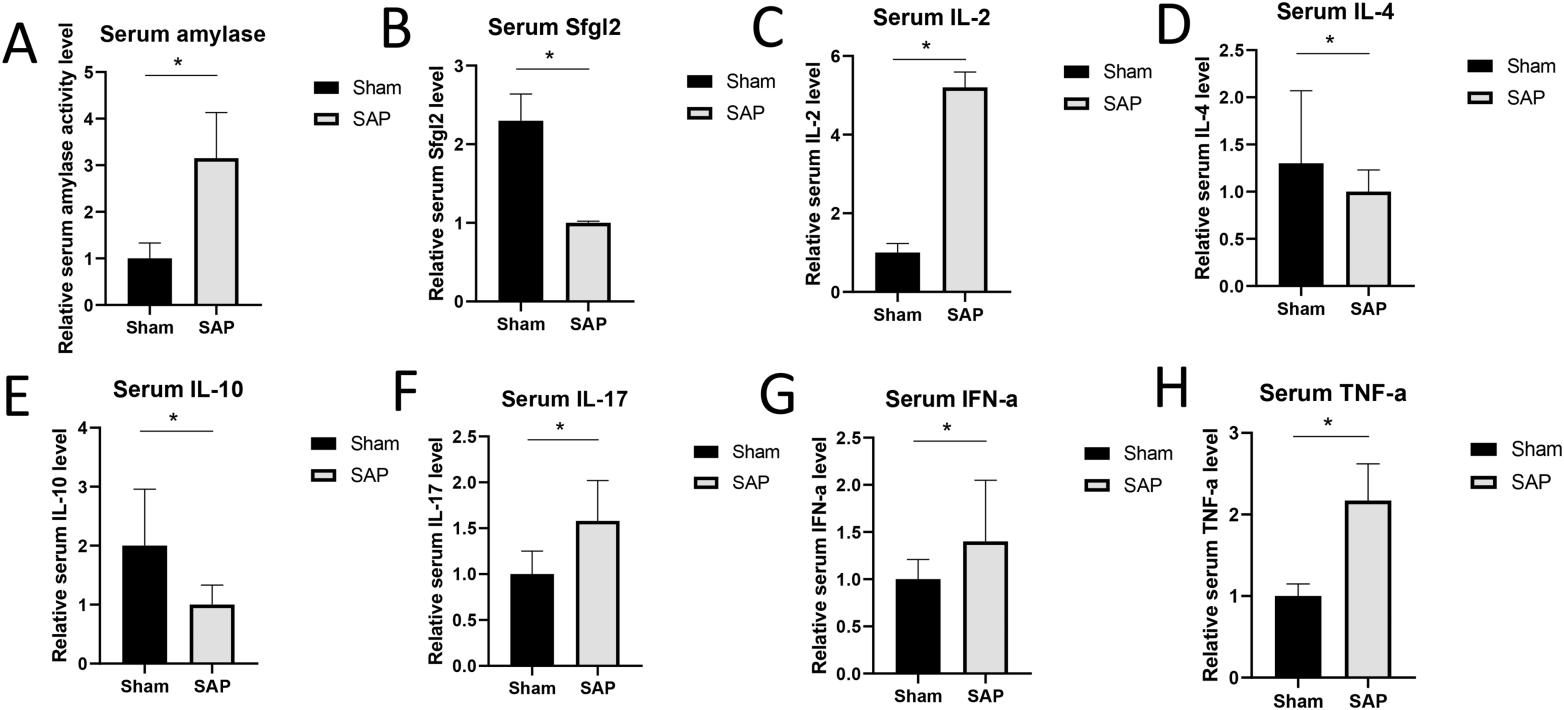
SAP induction lowered the serum Sfgl2 level and changed the levels of soluble inflammatory mediators in mouse blood. (A–H) At 4 h after surgery, the relative serum levels of amylase (A), Sfgl2 (B), IL‐2 (C), IL‐4 (D), IL‐10 (E), IL‐17 (F), IFN‐α (G), and TNF‐α (H) were measured and compared between the Sham group and the SAP group. *n* = 5 mice per group, *p < 0.05, between the indicated groups.

To further reveal the link between Sfgl2 and immunoregulation in the development of SAP, we measured the concentrations of Sfgl2 and some soluble Treg/Th17‐related inflammatory factors in the mouse serum samples. First, we confirmed that the mice in the SAP group showed a significantly higher level of serum amylase (Figure 4A), which is a reliable and frequently used biochemical indicator of AP. Consistent with the lower level of *Sfgl* mRNA in the pancreatic tissues of the SAP mice, these mice also had a markedly downregulated level of serum Sfgl2 than the Sham mice (Figure 4B). Among all of the cytokines tested, SAP induction in our mouse model resulted in significantly reduced serum concentrations of IL‐4 (Figure 4D) and IL‐10 (Figure 4E), and significantly elevated plasma levels of IL‐2 (Figure 4C), IL‐17 (Figure 4F), IFN‐α (Figure 4G), and TNF‐α (Figure 4H). Therefore, SAP induction led to a reduced Sfgl2 concentration and altered levels of inflammatory mediators in the mouse serum.

### 3.4 Bone Marrow–Derived DCs From SAP Mice Showed Significantly Reduced NF‐κB/p65 Nuclear Translocation and Enhanced Activation

**FIGURE 5:**
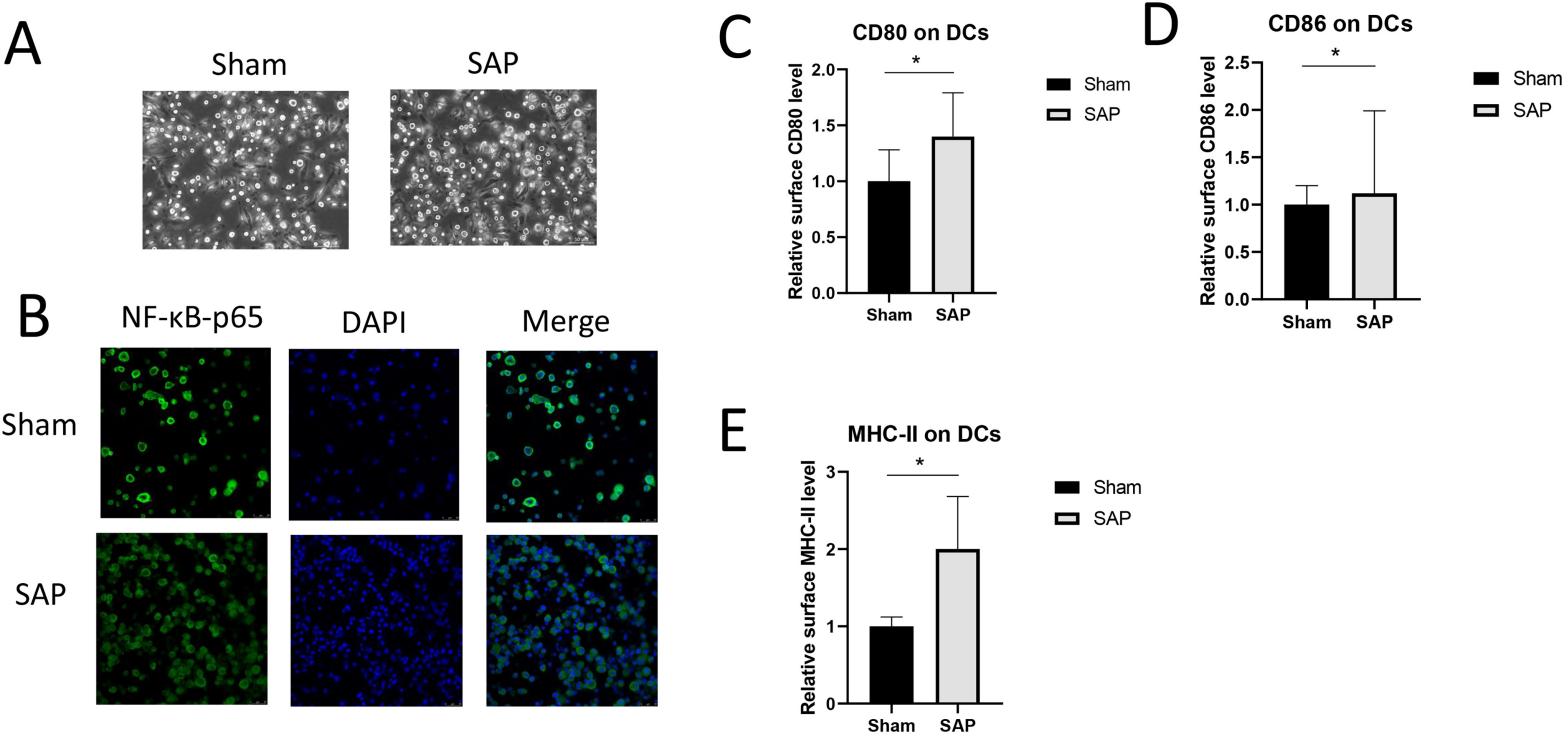
SAP induction in mice changed the NF‐κB/p65 nuclear translocation and activation status of bone marrow–derived DCs. (A) Bone marrow cells were isolated from mice at 4 h after surgery, and the bone marrow–derived DCs were enriched and purified after induction in culture medium containing IL‐4 and GM‐CSF. Representative images show the cellular morphology of DCs in the Sham and SAP groups. Magnification, 20×. (B) Representative images of immunofluorescence staining show the changes in nuclear translocation of NF‐κB‐p65 in the bone marrow–derived DCs from the Sham and SAP groups. Magnification, 20×. (C–E) The relative surface levels (percentages of positively stained cells among the total DCs) of DC activation markers including CD80 (C), CD86 (D), and MHC‐II (E) on bone marrow–derived DCs from the Sham and SAP groups were compared. *n* = 5 mice per group, *p < 0.05, between the indicated groups.

DCs are the master regulators of the immune responses and play a critical role in regulating the Treg and Th17 adaptive immune responses [31]. Thus, we further investigated how SAP mice with a lower level of Sfgl2 were affected in terms of the maturation and activation of DCs. We differentiated and isolated CD11c^+^ DCs from the bone marrow of mice in the Sham and SAP groups as well as validated them on the basis of their cell morphology (Figure 5A). As a pivotal mediator of inflammatory responses, NF‐κB modulates many aspects of innate and adaptive immune functions [32, 33]. The nuclear translocation of p65, a subunit of NF‐κB, was used to evaluate the activation of our isolated DCs. As shown by immunofluorescence staining in Figure 5B, the DCs from the Sham group exhibited more prominent NF‐κB/p65 nuclear translocation than those from the SAP group. However, the DCs generated from the SAP mice exhibited a significantly higher surface expression of activation markers, including CD80 (Figure 5C), CD86 (Figure 5D), and MHC‐II (Figure 5E), suggesting that SAP induction is associated with accelerated DC maturation. Collectively, these results indicate that the SAP‐induced low level of Sfgl2 could lead to potentially premature DCs with dysregulated NF‐κB/p65 translocation.

## 4 Discussion

Early pathology of AP with autodigestion of the pancreatic tissue results in the release of various proinflammatory cytokines and the development of pancreatic inflammation [2, 8, 14]. Meanwhile, SAP is related to tremendous systemic inflammation that is characterized by the release of multiple soluble inflammatory factors [15, 34]. The complex pathogenesis of SAP involves an intricate immune response, with different T‐cell subsets (such as Th1, Th2, Th17, and Treg cells) playing critical roles and contributing distinctly in the disease development processes [15, 34]. The balance between Th17 cells and Treg cells plays critical roles in maintaining immune tolerance, and the break of this balance has been implicated in the pathophysiology of SAP [19]. Here, using a taurocholate‐induced mouse model of SAP, we identified that SAP induction resulted in a lower local ratio of Th17/Treg in pancreatic tissue and a higher ratio of Th17/Treg in peripheral blood. In addition, SAP was associated with a reduced expression of Sfgl2 in pancreatic tissue, a downregulated serum level of Sfgl2, and significantly altered serum concentrations of multiple inflammation‐related cytokines. Furthermore, bone marrow–derived DCs from SAP mice demonstrated dysregulated NF‐κB/p65 nuclear translocation and aberrant activation. Our results suggest that the accumulation of anti‐inflammatory effects and the occurrence of immunosuppression co‐exist in the development of SAP.

Growing evidence has confirmed that Th17 cells are pathogenic in several autoimmune disease mouse models such as experimental allergic encephalomyelitis and collagen‐induced arthritis [35]. Notably, as they participate in inflammatory reactions, Th17 cells also have been reported to exert an essential role in the pathogenesis of autoimmune pancreatitis [36]. In our murine SAP model, we found that both the blood samples and pancreatic tissues from SAP mice demonstrated a higher frequency of Th17 cells than those from the control Sham mice, implicating that Th17 cells might play a broad role both locally and in the whole body during the initiation of SAP. Consistent with our observation, Guo et al. [19] found that patients with SAP with multiple organ dysfunction had a higher Th17 percentage than the healthy group and the SAP group with single organ dysfunction. Moreover, further subgroup analysis identified that the nonsurvival group showed a significantly higher blood Th17 percentage than the survival group [19]. Therefore, Th17‐mediated immune dysregulation can be identified in both the murine SAP model and patients with SAP. In addition, our results, together with those of others, suggest that the percentage of Th17 cells in the peripheral blood might be a valuable parameter for predicting disease severity and the prognosis of patients with SAP.

It is worth noting that the pancreatic tissues from the SAP mice exhibited a profoundly higher (~20‐fold higher) abundance of Treg cells among the total CD4^+^ T cells than those from the Sham mice. However, the SAP mice had a significantly lower Treg percentage in the blood than the Sham mice, thus resulting in a dramatic conversion from a high Th17/Treg ratio in the pancreas to a low Th17/Treg ratio in the peripheral blood. This conversion highlighted the role of Treg cells in anti‐inflammatory reactions, as local inflammation of the pancreatic tissue during SAP initiation might recruit more Treg cells from the peripheral blood to more effectively suppress the overactivated immune responses. The mobilization of Treg cells upon pancreatic injury indicates that Treg cells play a crucial role in the counterbalance against SIRS. In line with this intriguing finding, in an experimental mouse model of partial duct ligation‐induced AP using C57BL/6 mice, Glaubitz et al. [37] reported that Treg depletion enhances the proinflammatory response. Moreover, Li et al. [38] found that the number of Treg cells continuously increases during AP progression, which might be related to immune suppression, a characteristic of compensatory anti‐inflammatory response syndrome. They also proposed that immunosuppression events, including the shift of the Th1/Th2 balance toward a Th2 response, increased Treg cells and that the related inflammatory cytokines contributed to the complex process of inflammation and infection associated with gut mucosal barrier dysfunction in patients with early SAP [38]. Collectively, a previous study of patients with SAP and the current investigation using a murine SAP model both validated that the Th17/Treg imbalance exists in SAP induction and that Treg cells play an essential role in counterbalancing against local inflammation in the pancreas.

Sfgl2 is highly expressed by CD4^+^CD25^+^ Treg cells and is considered as a common effector molecule of many classes of Treg cells [23]. Functionally distinct from the membrane‐bound Fgl2, Sfgl2 possesses immunomodulatory activity and contradictory properties in tissue injuries [20]. We identified a lower blood Treg percentage and a lower concentration of serum Sfgl2 in the SAP group than the Sham group. Additionally, pancreatic tissues from the SAP mice also displayed lower expression levels of both Foxp3 mRNA and Sfgl2 mRNA, further implying that Treg cells are the major source of Sfgl2. Of note, the pancreatic tissues from the SAP mice showed a higher Treg percentage but a lower Foxp3 mRNA level than those from the Sham mice, probably due to the discrepancy in processing tissue samples for flow cytometry and qPCR as well as the possible large difference in the total numbers of Treg cells between the two groups. Previous studies have revealed a higher expression level of Fgl2 in blood cells and the pancreas during taurocholate‐induced AP progression in rats [26] as well as a higher Fgl2 expression in the blood cells of patients with SAP [25], compared with the control groups. Evidently, Sfgl2 exhibited a reversed expression level pattern upon SAP induction as Fgl2, which is most likely due to their distinct functions and sources: Fgl2 is expressed on the surface of macrophages or endothelial cells and cleaves prothrombin into thrombin to exert procoagulant activity, eventually leading to microthrombosis in the microvessels of pancreatic tissues, whereas Sfgl2 predominately originates from Treg cells and possesses immunomodulatory activity during pancreatic injury. Thus, further investigation on therapies targeting Fgl2, such as a neutralizing antibody or genetic therapy, should take into consideration the drastically different functions of Fgl2 and Sfgl2 in humans.

Sfgl2 has been reported to exert a multimodal role in the immune system and functions as a crucial immunosuppressive effector cytokine of Treg cells to suppress the production of Th1 cytokines (such as IL‐1β, IL‐2, IL‐12, TNF‐α, and IFN‐γ) while enhancing Th2 cytokines (such as IL‐4, IL‐5, and IL‐10) [20, 23]. Consistent with this notion, we found that a lower serum level of Sfgl2 in the SAP mice was associated with higher levels of serum IL‐2, IFN‐α, and TNF‐α as well as lower levels of serum IL‐4 and IL‐10. In addition, as the effector cytokine of Th17 cells, IL‐17 showed a higher serum level in SAP mice, which is in line with the higher Th17 percentage in both blood and pancreatic tissue upon SAP induction in our murine model. This finding also supports the previous proposal that IL‐17 is a predictive marker of early forms of SAP and that its concentration is correlated with the risk of organ dysfunction [39, 40]. Moreover, as an important mediator of Treg suppression, the anti‐inflammatory cytokine IL‐10 has been reported to provide Treg cells with the ability to suppress pathogenic Th17 cell responses [41]. The SAP‐associated lower serum level of IL‐10 might be caused by the recruitment and accumulation of IL‐10 in local organs such as the injured pancreas, where Treg cells are mobilized to fight against inflammation. Furthermore, exogenous Sfgl2 has been shown to lead to the reduced expression of CD80 and MHC‐II on bone marrow–derived DCs, thus rendering profound effects on suppressing T‐cell immunity of the adaptive immune system [42]. We found that bone marrow–derived DCs from the SAP mice showed markedly reduced NF‐κB/p65 nuclear translocation and enhanced activation, with higher expression levels of CD80, CD86, and MHC‐II. It is plausible that the SAP‐associated low level of Sfgl2 contributed to the dysregulation of DC maturation. However, as SAP runs a biphasic course and a transition from a proinflammatory response to an anti‐inflammatory response occurs [3], further investigation with monitoring Sfgl2 production during the whole course of DC generation from the bone marrow is necessary to understand the role of Sfgl2 in immunosuppression and modulating DC maturation.

Our study identified Sfgl2 as an SAP‐related biomarker and highlighted that the Th17/Treg imbalance exists in SAP induction, which contributed significantly to local inflammation in the pancreas. These findings suggest the potential of using exogenous Sfgl2 protein and other immunomodulators targeting the correction of the Th17/Treg imbalance to treat patients with SAP. Nevertheless, there are several limitations of this study that must be mentioned. First, as the results of this study were elicited only in taurocholate‐induced C57BL/6J mice, it is worth testing whether the conclusions remain the same in other SAP animal models with different strains and other approaches of modeling. However, as discussed above, our results support the previous findings that the accumulation of anti‐inflammatory effects and the occurrence of a Th17/Treg imbalance co‐exist in the development of SAP in both humans and mice. Second, female mice were used in this study because this could substantially exclude fight injury‐associated inflammation that is common in male mice. However, the physiological variability linked with the estrous cycle of female rodents is another concern, and gender bias‐related biological effects due to the hormonal and genetic intrinsic differences between female and male animals should be taken into consideration for results interpretation. Therefore, additional studies using a large number of animals of both genders (control males vs. experimental males and control females vs. experimental females) should be performed to reduce potential gender bias‐related biological effects and further consolidate our conclusions.

## 5 Conclusions

In summary, using a taurocholate‐induced mouse model of SAP, we revealed that T lymphocyte‐dependent immune dysfunction, such as an imbalanced Th17/Treg ratio, existed upon SAP initiation and that Sfgl2 had a low expression level in both the serum and pancreatic tissue. The downregulated Sfgl2 expression was accompanied with higher levels of Th1 cytokines (IL‐2 and TNF‐α) and lower levels of Th2 cytokines (IL‐4 and IL‐10) in the serum from SAP mice as well as dysregulated DC maturation. Therefore, our understanding of the Th17/Treg imbalance and inflammatory responses in the SAP murine model provides critical insights for the development of effective immunotherapies to enhance the outcomes of patients with SAP.

## Author Contributions

Y.H., J.D., and Y.C. conceived and designed the research. J.D., Y.C., and Q.W. collected data and conducted research. Y.H., X.Y., and H.H. analyzed and interpreted data. Y.H. wrote the initial draft. J.D. and X.Y. revised the manuscript. X.Y. had primary responsibility for the final content. All authors read and approved the final version of the manuscript.

## Ethics Statement

All experiments involving the use of animals were approved by the Experimental Animal Ethics Committee of Risai Biomedical Technology Co., Ltd. (approval No. 20201110012) and were carried out in accordance with the Guide for the Care and Use of Laboratory Animals (eighth edition) from the National Research Council (US) Committee. All methods were reported in accordance with the ARRIVE guidelines (https://arriveguidelines.org) for the reporting of animal experiments.

## Consent

The authors have nothing to report.

## Conflicts of Interest

The authors declare no conflicts of interest.

## Data Availability

The datasets used and/or analyzed during the current study are available from the corresponding author upon reasonable request.
